# Immunologic and virological response to ART among HIV infected individuals at a tertiary hospital in Ghana

**DOI:** 10.1186/s12879-018-3142-5

**Published:** 2018-05-21

**Authors:** Dorcas Obiri-Yeboah, Faustina Pappoe, Ibrahim Baidoo, Francis Arthur, Anna Hayfron-Benjamin, Samuel Essien-Baidoo, Godwin Kwakye-Nuako, Stephen Ayisi Addo

**Affiliations:** 1 Department of Microbiology and Immunology, School of Medical Sciences, University of Cape Coast, Cape Coast, Ghana; 2 Public Health Unit, Cape Coast Teaching Hospital, Cape Coast, Ghana; 3 Microbiology Unit, Cape Coast Teaching Hospital, Cape Coast, Ghana; 4 Department of Maternal and Child Health, School of Nursing and Midwifery, University of Cape Coast, Cape Coast, Ghana; 5 Department of Laboratory Technology, University of Cape Coast, Cape Coast, Ghana; 6 Department of Biomedical Sciences, University of Cape Coast, Cape Coast, Ghana; 7 National AIDS/STI Control Program, Ghana Health Services, Accra, Ghana

**Keywords:** Viral load, HIV, Antiretroviral therapy, Ghana, CD4

## Abstract

**Background:**

The need to study the outcome of Antiretroviral Therapy (ART) among Human Immunodeficiency Virus (HIV) infected individuals in Ghana, a sub-Saharan African country crucial in the era of the “Treat All” policy. The aim of this study was to analyze selected determinants of immunological and virological response to ART among HIV infected individuals in a tertiary facility in Cape Coast, Ghana.

**Methods:**

An analytical cross sectional study with a retrospective component was conducted in the Cape Coast Teaching Hospital (CCTH), Central Region. Clients aged 18 years and above attending the HIV Clinics for ART and who were on ART for 6 months or more were recruited. The viral loads, CD4 count and other socio-demographic data were analyzed using STATA version 13 (STATA Corp, Texas USA). Descriptive analysis was done and presented with appropriate measures of central tendencies. In addition, bivariate and multivariate analysis was carried out with *p value* of 0.05 interpreted as evidence of association between variables.

**Results:**

A total of 440 participants were included in this study with a mean age of 45.5 (±11.6) years. The mean CD4 count at baseline, 6 months on ART and currently at study recruitment were 215.1 cells/mm^3^ (±152.6), 386.6 cells/mm^3^ (±178.5), and 579.6 cells/mm^3^ (±203.0) respectively. After 6 months and 12 months on ART, the number who had achieved viral copies < 1000/ml were 149 (47.0%) and 368 (89.6%) respectively. There was strong evidence of an association between having CD4 count < 350 cells/mm^3^ after 6 months on ART and having a diagnosis of tuberculosis since HIV diagnosis (aOR 8.5, 95% CI 1.1–73.0, *p* = 0.05) and clients having plasma viral load > 1000 copies/ml after 6 months on ART (aOR 2.0, 95% CI 1.2–3.2, *p* = 0.01).

**Conclusion:**

There was good response to ART among clients, high virological suppression and immunological recovery hence low rates of change to second line ART regimen in this cohort studied. With strict adherence to the national policy on HIV testing, management of positive clients and full implementation of the “Treat All” policy, Ghana could achieve, if nothing at all, the third “90, 90, 90” target by 2020.

**Electronic supplementary material:**

The online version of this article (10.1186/s12879-018-3142-5) contains supplementary material, which is available to authorized users.

## Background

Human Immunodeficiency Virus (HIV) infection is still a major public health problem worldwide. Sub-Sahara Africa is home to at least 23 million infected people, most of whom report late to clinics for treatment [1, 2]. HIV infects and gradually depletes immune cells mainly CD4 lymphocytes which are key in cell-mediated response as well as contributing to humoral response against invaded pathogens [3]. As a result of immune destruction, severe morbidity and mortality which may also be associated with opportunistic infections are observed among those infected [4].

Globally, 20.9 million infected people were estimated to be receiving Anti-retroviral Therapy (ART) by June 2017 [5]. The antiretroviral (ARV) drugs, mostly used in combination of three, are targeted at inhibiting viral replication and subsequently maximizing reduction in viral load (VL) leading to recovery of immune function [3, 6]. Thus, ART reduces HIV associated morbidity and mortality and enables infected persons to enjoy quality and productive life.

Viral Load and CD4 counts are important markers for assessing treatment response and immune recovery in patients on ART. WHO recommends that VL and or CD4 testing where available should be done prior to ART, at 6 and 12 months after initiation of ART and then at least every 12 month once the patient becomes stable on ART [6]. This is to enable medical personnel to confirm suspected treatment failure detected clinically and to provide the necessary intervention such as adherence support and ART regimen switching [6]. Currently, treatment failure in Ghana is defined virologically (gold standard) as viral loads persistently more than 1000 copies of HIV RNAs/ml of blood plasma and or immunologically when CD4 counts are persistently at or below 250 cells/mm^3^ after 12 months on ART [7].

Recently, attention has been focused on immune recovery among Persons Living with HIV (PLHIV) on ART. Studies have highlighted the significance of ART and factors that may influence the outcomes of ART and immune recovery and these help to provide effective or better patient management and intervention. It has been reported that individuals who start ART at higher CD4 count baseline (≥300 cells/mm^3^) have their CD4 cell counts returning to nearly normal or normal (≥500 cells/mm^3^) than those who starts at lower baselines (≤200 cells/mm^3^) [8, 9]. Some studies have shown a link between viral suppression and improved immune response while on ART [10–12].

Little is known about the outcome of ART among HIV infected individuals in Ghana, a sub-Saharan African country with an estimated HIV prevalence of 1.47% [13]. There have been some studies focusing on the outcome for patients on specific ARVs and also the paediatric population [14, 15]. The aim of this study was to analyze the determinants of immunological and virological response to ART among HIV infected individuals in a tertiary facility in Cape Coast, Ghana.

## Methods

### Study design

An analytical cross sectional study with a retrospective component was conducted in the Cape Coast Teaching Hospital (CCTH), Central Region. HIV patients aged 18 years and above attending the HIV Clinics for ART from January to March 2017 were recruited after fulfilling the inclusion criteria and consenting to participate. Paediatric HIV patients, Adult PLHIV who have not been on ART for at least 6 months, clients with diabetes and women currently pregnant were excluded.

### Study area

The study was carried out at the HIV Clinics in CCTH, a referral hospital in Cape Coast Metropolis that provides services to patients within and beyond Central Region as a teaching hospital. The HIV clinic was the first to be set up in the region since 2006 and serves an average of 120 clients per week.

### Study population

A systematic sampling method was used to recruit the clients. All who met the inclusion criteria and were approached consented to be part of the study. The sample size for the study was calculated to be a minimum of 267 participants using confidence level of 95% and an error margin of 5% and the population on ART was 805 at the end of 2016. A maximum of 30 participants per clinic day were targeted so as to spread recruitment over a significant period. A total of 440 adult HIV positive clients who were on ART for 6 months or more were recruited. This was arrived at to target at least 50% of those on ART who met the inclusion criteria.

Socio-demographic characteristics were obtained from the patients using researcher administered questionnaires. Also, the participants’ clinical data were obtained from their clinical records under a data sharing agreement with the National AIDS/STI Control Programme (NACP). The information obtained were duration of HIV diagnosis, last WHO clinical stage before initiating ART, the use of co-trimoxazole prophylaxis, duration on ART, the ART regimen used, baseline, current CD4 count, plasma viral load after 6 months and if applicable, after 1 year on ART.

### Collection of blood samples

CD4+ T-cell determination was carried out at the Department of Microbiology Laboratory of the CCTH, Ghana using 50 μl of patients’ samples. The test is run using the fluorescence activated cell sorter (FACS) count flow cytometer (Becton Dickinson and Company, San Jose, USA) following the manufacturer’s instructions.

For viral load quantification, patients EDTA plasma (1000 μl) was used and run with the COBAS AmpliPrep/COBAS TaqMan HIV-1 test, v2.0 (Roche, Switzerland) with strict adherence to manufacturer’s instructions. This has an analytical sensitivity of 20 HIV-1 RNA copies/mL and specificity of 100%.

### Statistical analysis

Data was captured using Microsoft Excel and then cleaned and analyzed using STATA version 13 (STATA Corp, Texas USA). Descriptive analysis of socio-demographic and other characteristics data was done using appropriate measures of central tendencies. Bivariate and multivariate analysis was done and presented with *p* value of 0.05 interpreted as evidence of association between variables. Age; a recognised possible confounder and any variable with *p*-value ≤0.2 on bivariable analysis was included in the module for multivariate analysis.

### Ethical issues

This study was approved by the Institutional Review Board of the University of Cape-Coast (IRB-UCC) Cape Coast, Ghana. Permission to undertake the study at the HIV Clinics of the hospital was sought and granted by the hospital management. The participants enrolled in the study gave written informed consent after full explanation of the procedure in the language and/or dialect they best understand.

## Results

### Socio-demographic characteristics

A total of 440 participants were included in this study with a mean age of 45.5 (±11.6) years and majority (83.4%) being between 31 and 60 years. A total of 346 representing 78.6% were female and 60% were living in rural communities (*n* = 264), Table 1.

**Table 1:** Socio-demographic characteristics of 440 study participants

Variable	Mean (SD)/n (%)
Age (yrs.)	
Mean	45.5 (±11.6)
18–30	38 (8.6)
31–60	367 (83.4)
> 60	35 (8.0)
Gender	
Male	94 (21.4)
Female	346 (78.6)
Marital status	
Single	101 (22.9)
Married/cohabiting	179 (40.7)
Divorced/widowed	160 (36.4)
Educational Status	
None to primary	221 (50.2)
Up to secondary (senior high)	196 (44.5)
Tertiary	23 (5.2)
Employment	
Unemployed	67 (15.2)
Unskilled employment	346 (78.6)
Skilled employment	27 (6.1)
Place or Residence	
Urban	176 (40.0)
Rural	264 (60.0)

### HIV and ART related characteristics

A total of 432 (98.1%) of the participants had confirmed HIV-1 infection. Majority of 65.7% (*n* = 289) had been on ART for > 2 years and almost 95% (410) were still on first line ART regimen at the time of recruitment. The mean CD4 count at baseline, 6 months on ART and currently at study recruitment were 215.1 cells/mm^3^ (±152.6), 386.6 cells/mm^3^ (±178.5), and 579.6 cells/mm^3^ (±203.0) respectively. After 6 months and 12 months on ART, the number who had achieved viral copies < 1000/ml were 149 (47.0%) and 368 (89.6%) respectively, (Table 2).

**Table 2:** HIV associated clinical and laboratory characteristics of study participants

Variable	Mean (SD)/Median (IQR)/n (%)
Duration of HIV diagnosis (years)	
Median	2 (2,3)
< 2	83 (18.8)
2–5	172 (39.1)
> 5	185 (42.1)
HIV type	
1	432 (98.1)
1 & 2	8 (1.8)
Last WHO clinical stage before initiating ART	
1	21 (4.7)
2	101 (23.0)
3	249 (56.6)
4	69 (15.7)
History of tuberculosis since HIV diagnosis	
Yes	13 (2.9)
No	427 (97.1)
Duration on ART (years)	
< 1	82 (18.6)
1–2	69 (15.7)
> 2	289 (65.7)
ART regimen	
First line regimen	410 (93.2)
Second line regimen	30 (6.8)
CD4 count cells/mm^3^	
Baseline Mean (*N* = 414)	215.1 (±152.6)
Mean after 6 months on ART (*N* = 376)	386.6 (±178.5)
Current Mean (*N* = 396)	579.6 (±203.0)
Plasma viral load after 6 months on ART, copies/ml(*N* = 317)	
Median	1080 (198, 2598)
0	2 (0.6)
1–999	147 (46.4)
≥ 1000	168 (53.0)
Plasma viral load after 12 months on ART, copies/ml (*N* = 411)	
Median	90 (20, 328)
0	85 (20.7)
1–999	283 (68.9)
≥ 1000	43 (10.5)

**Fig. 1:**
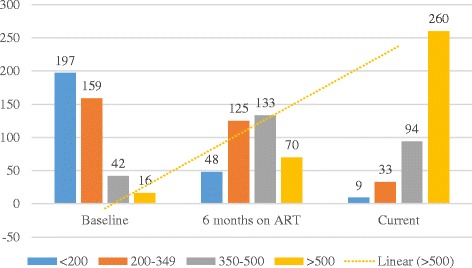
CD4 count values (cells/mm^3^) at baseline, 6mths on ART and current at recruitment

**Fig. 2:**
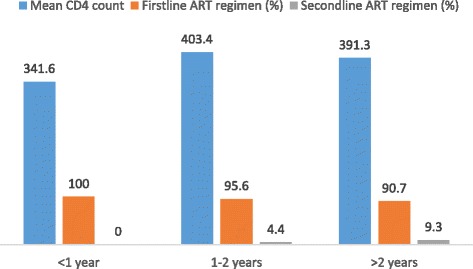
Current mean CD4 count (cells/mm^3^) and ART regimen (%) presented according to duration on ART

The number of clients with CD4 counts < 200 cells/mm^3^ decreased from 197 at baseline to 48 after 6 months on ART and to 9 at recruitment into this study (Fig. 1). Among participants who were on ART for < 1 year, 1–2 years and > 2 years, the proportion who were on second line ART regimen at the time of recruitment increased progressively from 0, 4.4 and 9.3% respectively (Fig. 2).

### Risk factor analysis

There was strong evidence of an association between having CD4 count < 350 cells/mm^3^ after 6 months on ART and being involved in a skilled employment (aOR 0.2, 95% CI 0.1–0.7, *p* = 0.01), having a diagnosis of tuberculosis since HIV diagnosis (aOR 8.5, 95% CI 1.1–73.0, *p* = 0.05) and clients having plasma viral load > 1000 copies/ml after 6 months on ART (aOR 2.0, 95% CI 1.2–3.2, *p* = 0.01), Table 3.

**Table 3:** Bivariate and multivariate analysis for participants with CD4 < 350 cells/mm^3^ after 6 months on ART (*N* = 173)

Variable	CD4 < 350, {n (%)}	OR (95% CI)	*P* value	^a^aOR (95% CI)	*P* value
Age					
18–30	13 (7.5)	–			
31–60	144 (83.2)	1.1 (0.5–2.3)	0.81	1.2 (0.5–2.9)	0.76
> 60	16 (9.3)	1.5 (0.5–4.1)	0.44	1.3 (0.4–5.6)	0.48
Gender					
Male	44 (25.4)	–			
Female	129 (74.6)	0.8 (0.4–1.1)	0.12	0.7 (0.4–1.3)	0.33
Employment					
Unemployed	34 (19.7)	–			
Unskilled employment	129 (74.6)	0.6 (0.3–0.9)	0.04	0.6 (0.3–1.2)	0.16
Skilled employment	10 (5.8)	0.4 (0.2–1.2)	0.09	0.2 (0.1–0.7)	***0.01***
Place of residence					
Urban	56 (32.4)	–			
Rural	117 (67.6)	1.7 (1.1–2.5)	0.02	1.5 (0.9–2.5)	0.11
WHO clinical Stage before starting ART					
1 & 2	40 (23.1)	–			
3 & 4	133 (76.9)	1.4 (0.9–2.2)	0.16	1.2 (0.7–2.3)	0.36
Tuberculosis Diagnosis					
No	165 (95.4)	–			
Yes	8 (4.6)	2.4 (0.7–8.2)	0.15	8.5 (1.1–73.0)	***0.05***
ART regimen					
First line	156 (90.2)	–			
Second line	17 (9.8)	1.9 (0.9–4.2)	0.11	1.6 (0.7–3.8)	0.29
Plasma viral load after 6 months on ART (*N* = 139)					
≤ 1000 copies/ml	53 (38.1)	–			
> 1000 copies/ml	86 (61.9)	2.2 (1.4–3.6)	0.001	2.0 (1.2–3.2)	***0.01***
Educational level					
None to primary	85 (49.1)	–			
Up to secondary (senior high)	79 (45.7)	1.1 (0.8–1.7)	0.52		
Tertiary	9 (5.2)	0.9 (0.3–2.1)	0.73		
HIV type					
1 alone	168 (97.1)	–			
1 & 2	5 (2.9)	2.0 (0.5–8.5)	0.34		

*P*-values in bold and italicized shows variables with evidence of association in bi-variate and multivariate analysis

^a^model included age, gender, occupation, residence, WHO clinical stage at starting ART, History of TB diagnosis since starting ART, ART regimen and plasma viral load after 6 months on ART

## Discussions

The main goal of ART for people living with HIV (PLHIV) is to achieve virological suppression and immune system recovery. This would ensure quality healthy living and continuous contribution to families, community and country as a whole. Such viral suppression would also very importantly reduce the risk of HIV transmission to sexual partners and from mother to child [16–18]. Many factors affect the response to ART and this study aimed at determining these factors among this study population after a minimum of 6 months on ART.

This study found that after 6 months and 12 months on ART, the number of participants who had achieved viral copies < 1000/ml rose from 149 (47.0%) to 368 (89.6%) respectively. In fact, 2 people (0.6%) had achieved complete viral suppression (undetectable viral copies) after 6 months on ART, but this number rose to 85 (20.7%) after 12 months. This virological suppression on first line ARVs is very encouraging and comes close to the new global UNAIDS target which indicates that by the year 2020, at least 90% of all clients initiated on ART should have achieved virological suppression after 12 months on ART [19]. WHO recommends that early initiation of ART in positive persons would improve response and help countries to most likely achieve the 2020 target for virological suppression [20]. It is noteworthy that, in this study as much as 318 (72.3%) of clients were initiated into ART when they already had WHO clinical stage 3 or 4 conditions. In a study in Ghana, Kwakye-Nuako et al. (2016) found that clients with CD4 count < 200 cells/mm^3^ had increased prevalence of diarrhoea causing opportunistic pathogens (WHO clinical Stage 4 pathogens) [21] and such conditions would influence the response to therapy. Ghana has currently adopted the “Treat All” policy which ensures that all confirmed HIV infected individuals in Ghana qualify for ART and efforts are to be made to initiate as soon as possible [7]. Immunologically, the mean CD4 count at baseline, 6 months on ART and at recruitment into this study were 215.1 cells/mm^3^, 386.6 cells/mm^3^, and 579.6 cells/mm^3^ respectively showing a progressive rise as expected. There was strong evidence of an association between having CD4 count < 350 cells/mm^3^ after 6 months on ART and having plasma viral load > 1000 copies/ml (aOR 2.0, 95% CI 1.2–3.2, *p* = 0.01). These findings agree with the knowledge that, viral suppression leads to immune recovery. Some studies looking at the relationship between CD4 count and virological suppression have found discordant relationship in some participants where virological suppression does not reflect in the rise in CD4 count. Casotti et al. (2011) attributed this to delays in initiating ART [22] while Kelly et al. (2016) in their systematic review on clinical outcomes, found increased mortality among clients with such discordant response [23].

In this study, the mean age of participants was 45.5 years (±11.6), this implies a relatively older population initiated on ART. In this study, age was not found to predict a client having CD4 count < 350 cells/mm^3^ after 6 months on ART on both bivariate and multivariate analysis. A study by Gezie et al. (2016) in Ethiopia found that younger age contributed to higher increment of CD4 count on ART [24]. Other studies have also been reported by studies in other settings [25] while others have found that in the long term older clients achieved similar virological suppression compared with younger clients [26]. Majority (346, 78.6%) of participants were female which is consistent of the gender distribution of the HIV population in Ghana and even globally. And in this study there was no evidence of an association between gender and having CD4 count < 350 cells/mm^3^ after 6 months on ART as also reported by other studies [27, 28]. In a large study conducted in Canada, Cescon et al. (2014) reported that women were at heightened risk of having poor response to ART but did conclude that this needs further research [29]. The fact is that these socio-demographic factors all influence adherence to ART and any factor which leads to poor or sub-optimum adherence would affect the response to therapy and these include employment status, the issue of disclosure to sexual partners etc. [30–32]. The association between tuberculosis and HIV is also well established and in this study there was strong evidence of an association between having CD4 count < 350 cells/mm^3^ after 6 months on ART and having a diagnosis of tuberculosis (aOR 8.5, 95% CI 1.1–73.0, *p* = 0.05). This association informed the policy which is also adhered to in Ghana, for the screening of PLHIV for tuberculosis. Such a strategy has been found to be cost effective [33] and would lead to treatment of both conditions thus improving outcome [34].

This study while looking at virological and immunological response to ART among this cohort, had the limitation of not assessing the level of adherence to ART among the partcipants and other factors like prescribing practice and commodity availability. Such information would have contributed important information to explain the level of response found. Despite these limitations, the study provide useful finding which can inform ART clinicians and policy makers on what might need to be done to help Ghana work towards the “90, 90, 90” targets particularly with respect to achieving virological suppression at 12 months on ART.

## Conclusion

In this tertiary facility, there was good response to ART among clients, high virological suppression and immunological recovery hence low rates of change to second line ART regimen in this cohort studied. This is very encouraging for the national control programme and shows that with strict adherence to the national policy on HIV testing, management of positive clients and full implementation of the “Treat All” policy, Ghana could achieve, if nothing at all, the third “90, 90, 90” target by 2020.

## Additional file

Additional file 1: Dataset. (XLSX 50 kb)

## Abbreviations

AIDS: Acquired Immunodeficiency syndrome
ART: Antiretroviral therapy
CCTH: Cape Coast Teaching Hospital
CD4: Cluster of Differentiation
CI: Confidence Interval
HIV: Human Immunodeficiency Virus
NACP: National AIDS Control Programme
OR: Odds Ratio
PLHIV: People Living With HIV
UCC: University of Cape Coast
VL: Viral Load
WHO: World Health Organization
